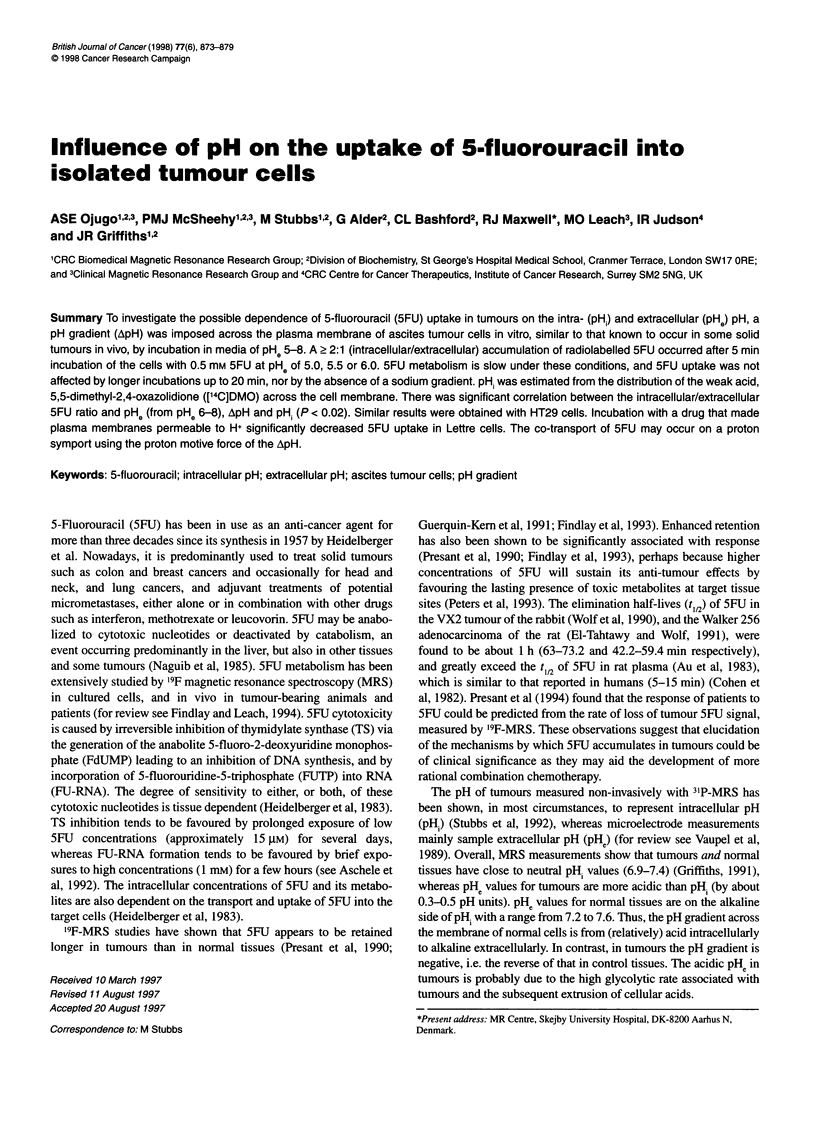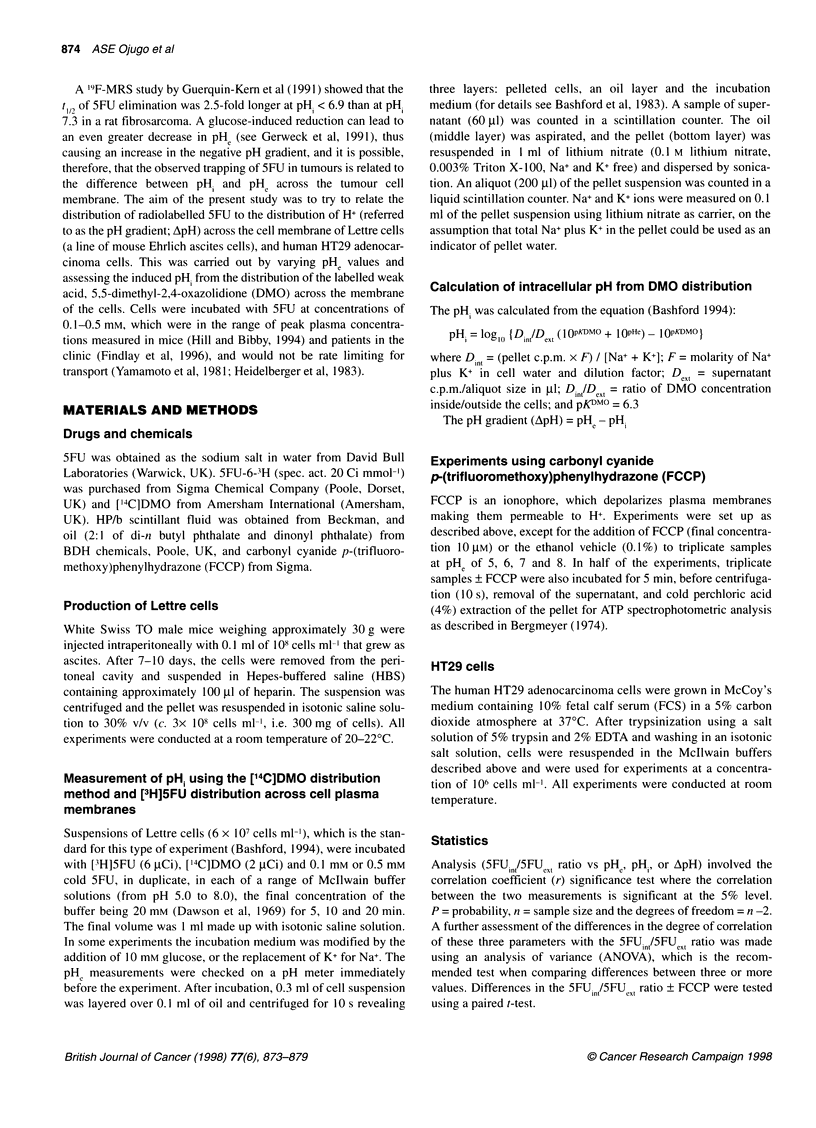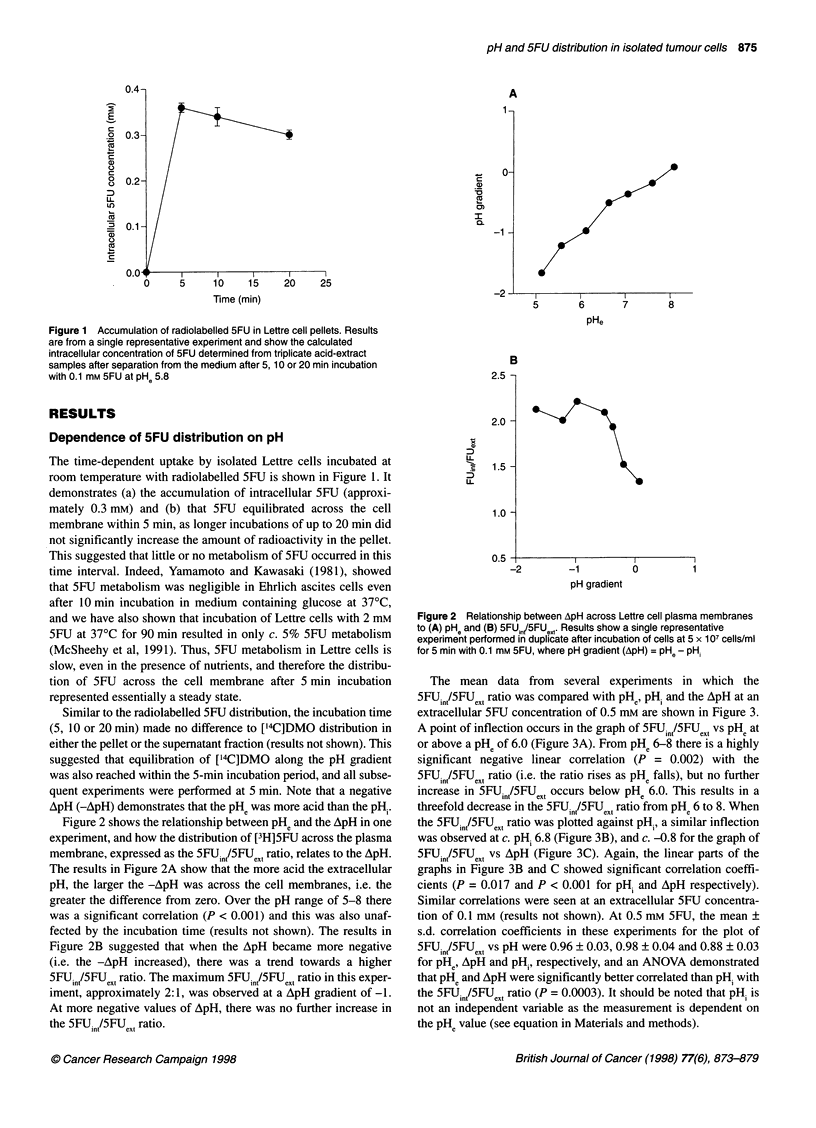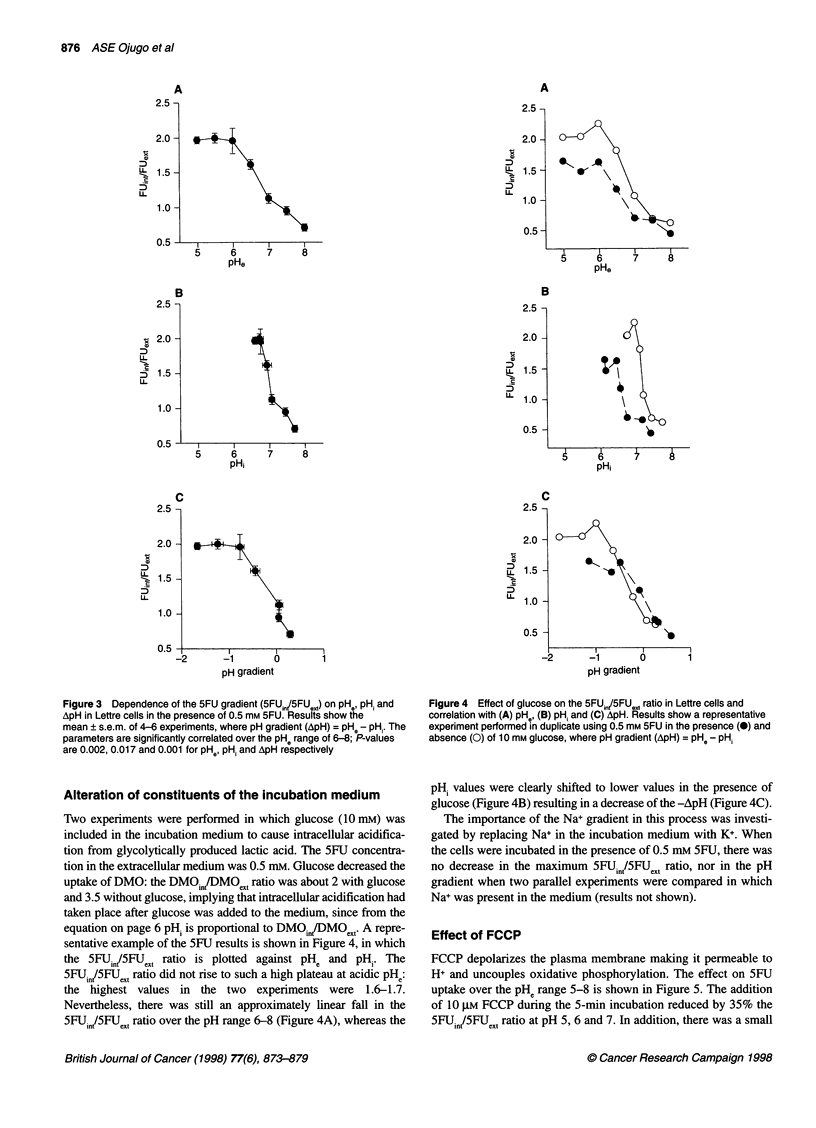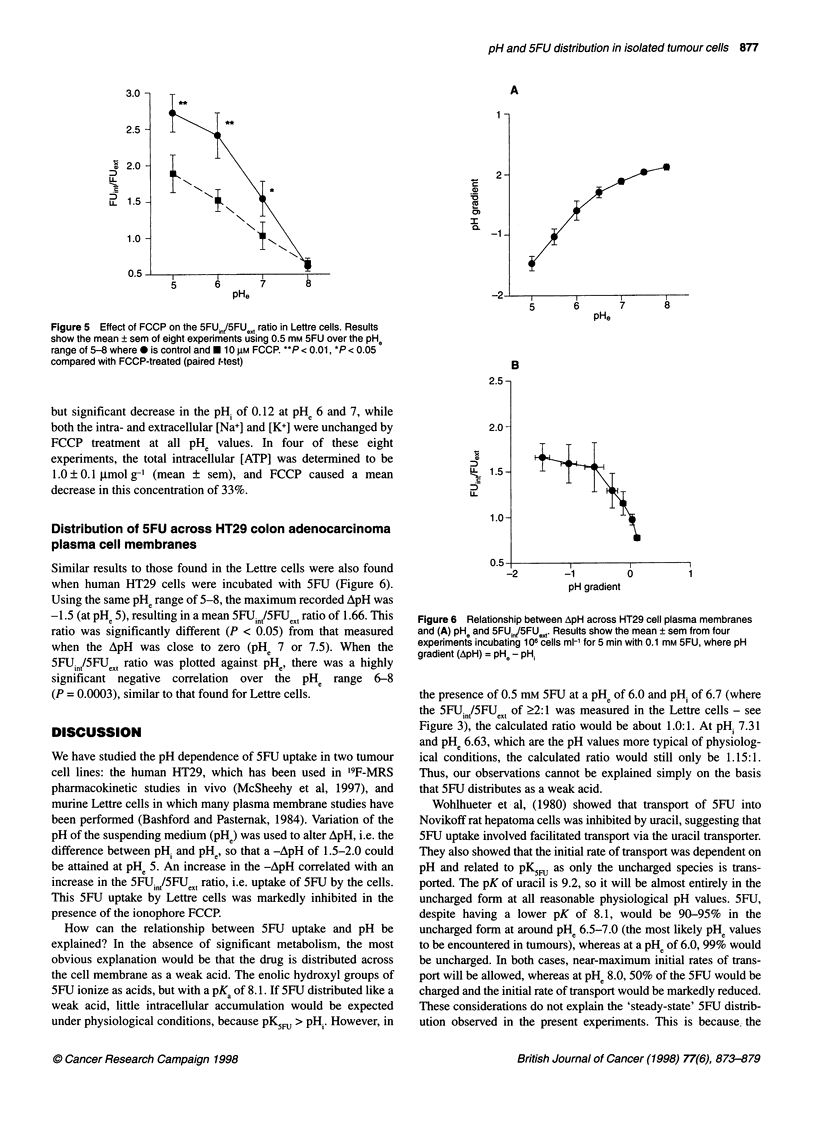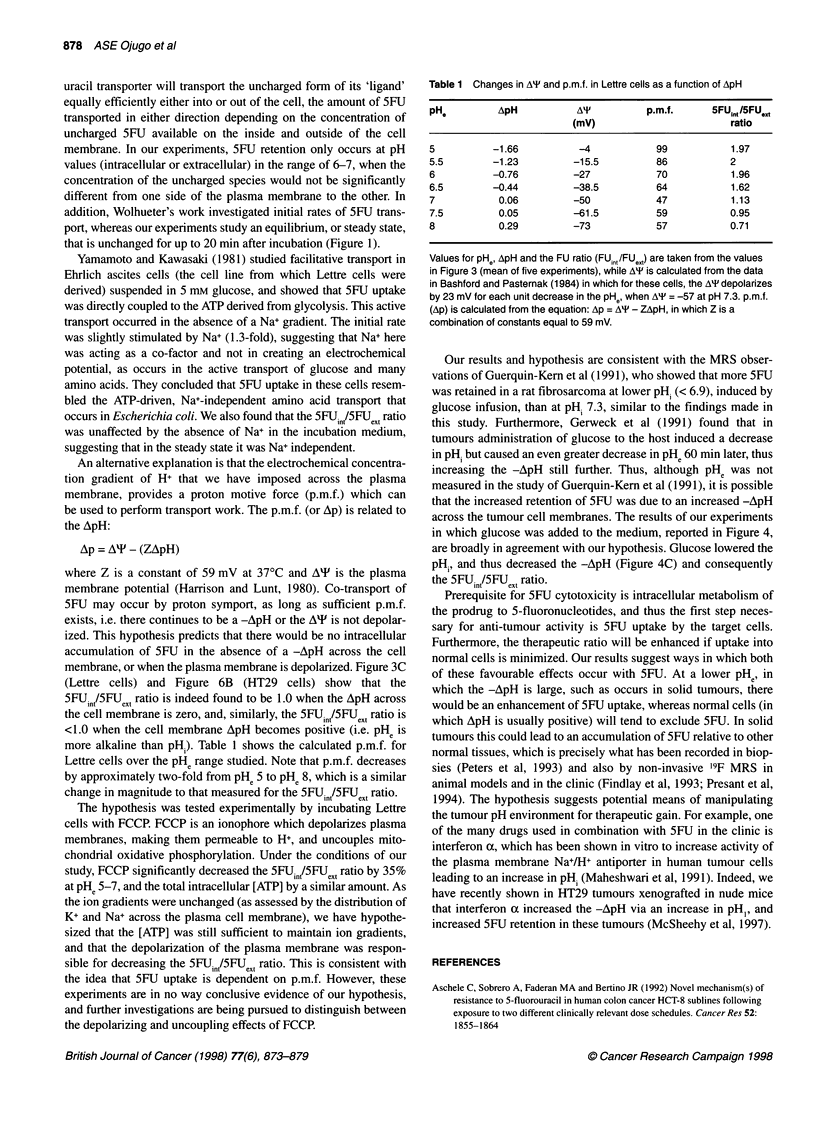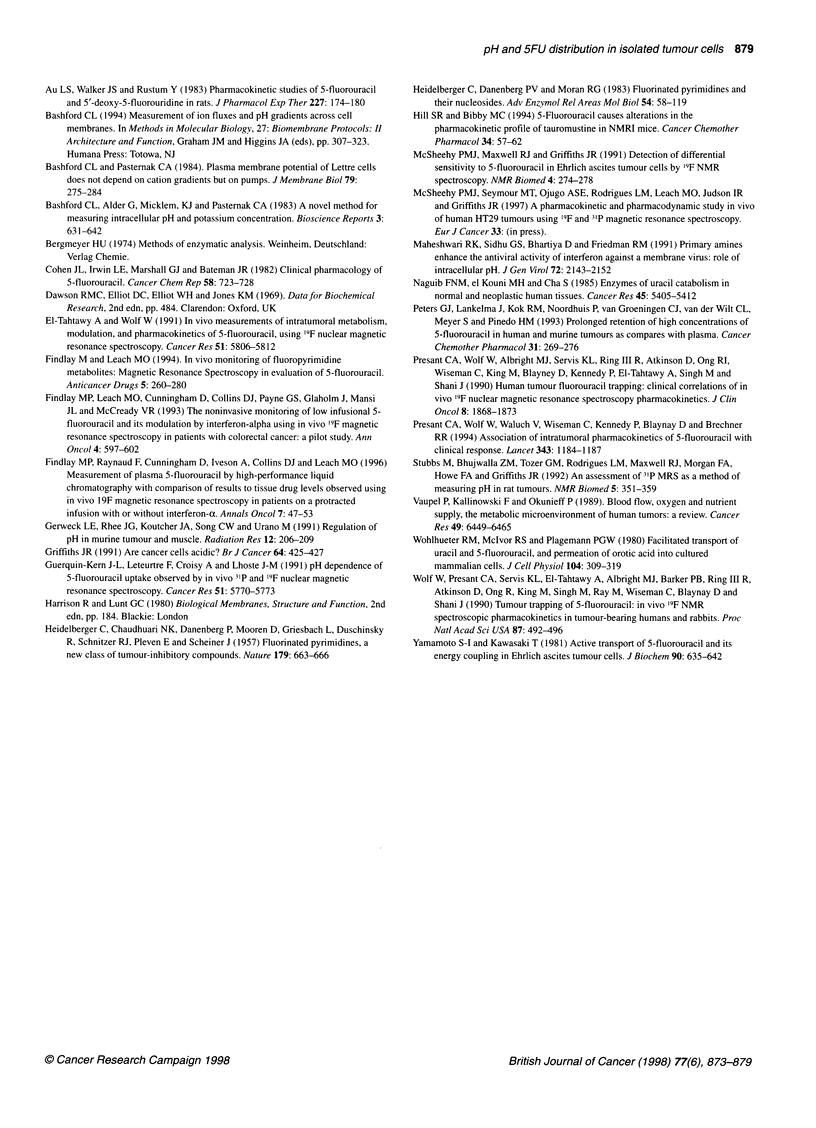# Influence of pH on the uptake of 5-fluorouracil into isolated tumour cells.

**DOI:** 10.1038/bjc.1998.144

**Published:** 1998-03

**Authors:** A. S. Ojugo, P. M. McSheehy, M. Stubbs, G. Alder, C. L. Bashford, R. J. Maxwell, M. O. Leach, I. R. Judson, J. R. Griffiths

**Affiliations:** Division of Biochemistry, St George's Hospital Medical School, London, UK.

## Abstract

To investigate the possible dependence of 5-fluorouracil (5FU) uptake in tumours on the intra- (pHi) and extracellular (pHe) pH, a pH gradient (deltapH) was imposed across the plasma membrane of ascites tumour cells in vitro, similar to that known to occur in some solid tumours in vivo, by incubation in media of PHe 5-8. A > or = 2:1 (intracellular/extracellular) accumulation of radiolabelled 5FU occurred after 5 min incubation of the cells with 0.5 mM 5FU at pHe of 5.0, 5.5 or 6.0. 5FU metabolism is slow under these conditions, and 5FU uptake was not affected by longer incubations up to 20 min, nor by the absence of a sodium gradient. pHi was estimated from the distribution of the weak acid, 5.5-dimethyl-2,4-oxazolidione ([14C]DMO) across the cell membrane. There was significant correlation between the intracellular/extracellular 5FU ratio and pHe (from pHe 6-8), deltapH and pHi (P < 0.02). Similar results were obtained with HT29 cells. Incubation with a drug that made plasma membranes permeable to H+ significantly decreased 5FU uptake in Lettre cells. The co-transport of 5FU may occur on a proton symport using the proton motive force of the deltapH.


British Journal of Cancer (1998) 77(6), 873–879
© 1998 Cancer Research Campaign

### Influence of pH on the uptake of 5-fluorouracil into isolated tumour cells

**ASE Ojugo[1,2,3], PMJ McSheehy[1,2,3], M Stubbs[1,2], G Alder[2], CL Bashford[2], RJ Maxwell*, MO Leach[3], IR Judson[4] and JR Griffiths[1,2]**

[1]CRC Biomedical Magnetic Resonance Research Group; [2]Division of Biochemistry, St George's Hospital Medical School, Cranmer Terrace, London SW17 0RE; and [3]Clinical Magnetic Resonance Research Group and [4]CRC Centre for Cancer Therapeutics, Institute of Cancer Research, Surrey SM2 5NG, UK

**Summary** To investigate the possible dependence of 5-fluorouracil (5FU) uptake in tumours on the intra- ($pH_i$) and extracellular ($pH_e$) pH, a pH gradient (ΔpH) was imposed across the plasma membrane of ascites tumour cells in vitro, similar to that known to occur in some solid tumours in vivo, by incubation in media of $pH_e$ 5–8. A ≥ 2:1 (intracellular/extracellular) accumulation of radiolabelled 5FU occurred after 5 min incubation of the cells with 0.5 mM 5FU at $pH_e$ of 5.0, 5.5 or 6.0. 5FU metabolism is slow under these conditions, and 5FU uptake was not affected by longer incubations up to 20 min, nor by the absence of a sodium gradient. $pH_i$ was estimated from the distribution of the weak acid, 5,5-dimethyl-2,4-oxazolidione ([$^{14}$C]DMO) across the cell membrane. There was significant correlation between the intracellular/extracellular 5FU ratio and $pH_e$ (from $pH_e$ 6–8), ΔpH and $pH_i$ ($P < 0.02$). Similar results were obtained with HT29 cells. Incubation with a drug that made plasma membranes permeable to $H^+$ significantly decreased 5FU uptake in Lettre cells. The co-transport of 5FU may occur on a proton symport using the proton motive force of the ΔpH.

**Keywords**: 5-fluorouracil; intracellular pH; extracellular pH; ascites tumour cells; pH gradient

5-Fluorouracil (5FU) has been in use as an anti-cancer agent for more than three decades since its synthesis in 1957 by Heidelberger et al. Nowadays, it is predominantly used to treat solid tumours such as colon and breast cancers and occasionally for head and neck, and lung cancers, and adjuvant treatments of potential micrometastases, either alone or in combination with other drugs such as interferon, methotrexate or leucovorin. 5FU may be anabolized to cytotoxic nucleotides or deactivated by catabolism, an event occurring predominantly in the liver, but also in other tissues and some tumours (Naguib et al, 1985). 5FU metabolism has been extensively studied by $^{19}$F magnetic resonance spectroscopy (MRS) in cultured cells, and in vivo in tumour-bearing animals and patients (for review see Findlay and Leach, 1994). 5FU cytotoxicity is caused by irreversible inhibition of thymidylate synthase (TS) via the generation of the anabolite 5-fluoro-2-deoxyuridine monophosphate (FdUMP) leading to an inhibition of DNA synthesis, and by incorporation of 5-fluorouridine-5-triphosphate (FUTP) into RNA (FU-RNA). The degree of sensitivity to either, or both, of these cytotoxic nucleotides is tissue dependent (Heidelberger et al, 1983). TS inhibition tends to be favoured by prolonged exposure of low 5FU concentrations (approximately 15 μM) for several days, whereas FU-RNA formation tends to be favoured by brief exposures to high concentrations (1 mM) for a few hours (see Aschele et al, 1992). The intracellular concentrations of 5FU and its metabolites are also dependent on the transport and uptake of 5FU into the target cells (Heidelberger et al, 1983).

$^{19}$F-MRS studies have shown that 5FU appears to be retained longer in tumours than in normal tissues (Presant et al, 1990; Guerquin-Kern et al, 1991; Findlay et al, 1993). Enhanced retention has also been shown to be significantly associated with response (Presant et al, 1990; Findlay et al, 1993), perhaps because higher concentrations of 5FU will sustain its anti-tumour effects by favouring the lasting presence of toxic metabolites at target tissue sites (Peters et al, 1993). The elimination half-lives ($t_{1/2}$) of 5FU in the VX2 tumour of the rabbit (Wolf et al, 1990), and the Walker 256 adenocarcinoma of the rat (El-Tahtawy and Wolf, 1991), were found to be about 1 h (63–73.2 and 42.2–59.4 min respectively), and greatly exceed the $t_{1/2}$ of 5FU in rat plasma (Au et al, 1983), which is similar to that reported in humans (5–15 min) (Cohen et al, 1982). Presant et al (1994) found that the response of patients to 5FU could be predicted from the rate of loss of tumour 5FU signal, measured by $^{19}$F-MRS. These observations suggest that elucidation of the mechanisms by which 5FU accumulates in tumours could be of clinical significance as they may aid the development of more rational combination chemotherapy.

The pH of tumours measured non-invasively with $^{31}$P-MRS has been shown, in most circumstances, to represent intracellular pH ($pH_i$) (Stubbs et al, 1992), whereas microelectrode measurements mainly sample extracellular pH ($pH_e$) (for review see Vaupel et al, 1989). Overall, MRS measurements show that tumours *and* normal tissues have close to neutral $pH_i$ values (6.9–7.4) (Griffiths, 1991), whereas $pH_e$ values for tumours are more acidic than $pH_i$ (by about 0.3–0.5 pH units). $pH_e$ values for normal tissues are on the alkaline side of $pH_i$ with a range from 7.2 to 7.6. Thus, the pH gradient across the membrane of normal cells is from (relatively) acid intracellularly to alkaline extracellularly. In contrast, in tumours the pH gradient is negative, i.e. the reverse of that in control tissues. The acidic $pH_e$ in tumours is probably due to the high glycolytic rate associated with tumours and the subsequent extrusion of cellular acids.

*Received 10 March 1997*
*Revised 11 August 1997*
*Accepted 20 August 1997*

*Correspondence to:* M Stubbs

**Present address:* MR Centre, Skejby University Hospital, DK-8200 Aarhus N, Denmark.

A $^{19}$F-MRS study by Guerquin-Kern et al (1991) showed that the $t_{1/2}$ of 5FU elimination was 2.5-fold longer at $pH_i < 6.9$ than at $pH_i$ 7.3 in a rat fibrosarcoma. A glucose-induced reduction can lead to an even greater decrease in $pH_e$ (see Gerweck et al, 1991), thus causing an increase in the negative pH gradient, and it is possible, therefore, that the observed trapping of 5FU in tumours is related to the difference between $pH_i$ and $pH_e$ across the tumour cell membrane. The aim of the present study was to try to relate the distribution of radiolabelled 5FU to the distribution of $H^+$ (referred to as the pH gradient; ΔpH) across the cell membrane of Lettre cells (a line of mouse Ehrlich ascites cells), and human HT29 adenocarcinoma cells. This was carried out by varying $pH_e$ values and assessing the induced $pH_i$ from the distribution of the labelled weak acid, 5,5-dimethyl-2,4-oxazolidione (DMO) across the membrane of the cells. Cells were incubated with 5FU at concentrations of 0.1–0.5 mM, which were in the range of peak plasma concentrations measured in mice (Hill and Bibby, 1994) and patients in the clinic (Findlay et al, 1996), and would not be rate limiting for transport (Yamamoto et al, 1981; Heidelberger et al, 1983).

### MATERIALS AND METHODS

### Drugs and chemicals

5FU was obtained as the sodium salt in water from David Bull Laboratories (Warwick, UK). 5FU-6-$^3$H (spec. act. 20 Ci mmol$^{-1}$) was purchased from Sigma Chemical Company (Poole, Dorset, UK) and [$^{14}$C]DMO from Amersham International (Amersham, UK). HP/b scintillant fluid was obtained from Beckman, and oil (2:1 of di-*n* butyl phthalate and dinonyl phthalate) from BDH chemicals, Poole, UK, and carbonyl cyanide *p*-(trifluoromethoxy)phenylhydrazone (FCCP) from Sigma.

### Production of Lettre cells

White Swiss TO male mice weighing approximately 30 g were injected intraperitoneally with 0.1 ml of $10^8$ cells ml$^{-1}$ that grew as ascites. After 7–10 days, the cells were removed from the peritoneal cavity and suspended in Hepes-buffered saline (HBS) containing approximately 100 μl of heparin. The suspension was centrifuged and the pellet was resuspended in isotonic saline solution to 30% v/v (*c.* 3× $10^8$ cells ml$^{-1}$, i.e. 300 mg of cells). All experiments were conducted at a room temperature of 20–22°C.

### Measurement of $pH_i$ using the [$^{14}$C]DMO distribution method and [$^3$H]5FU distribution across cell plasma membranes

Suspensions of Lettre cells (6 × $10^7$ cells ml$^{-1}$), which is the standard for this type of experiment (Bashford, 1994), were incubated with [$^3$H]5FU (6 μCi), [$^{14}$C]DMO (2 μCi) and 0.1 mM or 0.5 mM cold 5FU, in duplicate, in each of a range of McIlwain buffer solutions (from pH 5.0 to 8.0), the final concentration of the buffer being 20 mM (Dawson et al, 1969) for 5, 10 and 20 min. The final volume was 1 ml made up with isotonic saline solution. In some experiments the incubation medium was modified by the addition of 10 mM glucose, or the replacement of $K^+$ for $Na^+$. The $pH_e$ measurements were checked on a pH meter immediately before the experiment. After incubation, 0.3 ml of cell suspension was layered over 0.1 ml of oil and centrifuged for 10 s revealing three layers: pelleted cells, an oil layer and the incubation medium (for details see Bashford et al, 1983). A sample of supernatant (60 μl) was counted in a scintillation counter. The oil (middle layer) was aspirated, and the pellet (bottom layer) was resuspended in 1 ml of lithium nitrate (0.1 M lithium nitrate, 0.003% Triton X-100, $Na^+$ and $K^+$ free) and dispersed by sonication. An aliquot (200 μl) of the pellet suspension was counted in a liquid scintillation counter. $Na^+$ and $K^+$ ions were measured on 0.1 ml of the pellet suspension using lithium nitrate as carrier, on the assumption that total $Na^+$ plus $K^+$ in the pellet could be used as an indicator of pellet water.

### Calculation of intracellular pH from DMO distribution

The $pH_i$ was calculated from the equation (Bashford 1994):

$$pH_i = \log_{10} \{D_{int}/D_{ext}\,(10^{pK^{DMO}} + 10^{pHe}) - 10^{pK^{DMO}}\}$$

where $D_{int}$ = (pellet c.p.m. × $F$) / [$Na^+$ + $K^+$]; $F$ = molarity of $Na^+$ plus $K^+$ in cell water and dilution factor; $D_{ext}$ = supernatant c.p.m./aliquot size in μl; $D_{int}/D_{ext}$ = ratio of DMO concentration inside/outside the cells; and $pK^{DMO} = 6.3$

The pH gradient (ΔpH) = $pH_e - pH_i$

### Experiments using carbonyl cyanide *p*-(trifluoromethoxy)phenylhydrazone (FCCP)

FCCP is an ionophore, which depolarizes plasma membranes making them permeable to $H^+$. Experiments were set up as described above, except for the addition of FCCP (final concentration 10 μM) or the ethanol vehicle (0.1%) to triplicate samples at $pH_e$ of 5, 6, 7 and 8. In half of the experiments, triplicate samples ± FCCP were also incubated for 5 min, before centrifugation (10 s), removal of the supernatant, and cold perchloric acid (4%) extraction of the pellet for ATP spectrophotometric analysis as described in Bergmeyer (1974).

### HT29 cells

The human HT29 adenocarcinoma cells were grown in McCoy's medium containing 10% fetal calf serum (FCS) in a 5% carbon dioxide atmosphere at 37°C. After trypsinization using a salt solution of 5% trypsin and 2% EDTA and washing in an isotonic salt solution, cells were resuspended in the McIlwain buffers described above and were used for experiments at a concentration of $10^6$ cells ml$^{-1}$. All experiments were conducted at room temperature.

### Statistics

Analysis ($5FU_{int}/5FU_{ext}$ ratio vs $pH_e$, $pH_i$, or ΔpH) involved the correlation coefficient ($r$) significance test where the correlation between the two measurements is significant at the 5% level. $P$ = probability, $n$ = sample size and the degrees of freedom = $n$ –2. A further assessment of the differences in the degree of correlation of these three parameters with the $5FU_{int}/5FU_{ext}$ ratio was made using an analysis of variance (ANOVA), which is the recommended test when comparing differences between three or more values. Differences in the $5FU_{int}/5FU_{ext}$ ratio ± FCCP were tested using a paired $t$-test.

 © Cancer Research Campaign 1998

**Figure 1** Accumulation of radiolabelled 5FU in Lettre cell pellets. Results are from a single representative experiment and show the calculated intracellular concentration of 5FU determined from triplicate acid-extract samples after separation from the medium after 5, 10 or 20 min incubation with 0.1 mM 5FU at $pH_e$ 5.8

### RESULTS

### Dependence of 5FU distribution on pH

The time-dependent uptake by isolated Lettre cells incubated at room temperature with radiolabelled 5FU is shown in Figure 1. It demonstrates (a) the accumulation of intracellular 5FU (approximately 0.3 mM) and (b) that 5FU equilibrated across the cell membrane within 5 min, as longer incubations of up to 20 min did not significantly increase the amount of radioactivity in the pellet. This suggested that little or no metabolism of 5FU occurred in this time interval. Indeed, Yamamoto and Kawasaki (1981), showed that 5FU metabolism was negligible in Ehrlich ascites cells even after 10 min incubation in medium containing glucose at 37°C, and we have also shown that incubation of Lettre cells with 2 mM 5FU at 37°C for 90 min resulted in only *c.* 5% 5FU metabolism (McSheehy et al, 1991). Thus, 5FU metabolism in Lettre cells is slow, even in the presence of nutrients, and therefore the distribution of 5FU across the cell membrane after 5 min incubation represented essentially a steady state.

Similar to the radiolabelled 5FU distribution, the incubation time (5, 10 or 20 min) made no difference to [$^{14}$C]DMO distribution in either the pellet or the supernatant fraction (results not shown). This suggested that equilibration of [$^{14}$C]DMO along the pH gradient was also reached within the 5-min incubation period, and all subsequent experiments were performed at 5 min. Note that a negative ΔpH (–ΔpH) demonstrates that the $pH_e$ was more acid than the $pH_i$.

Figure 2 shows the relationship between $pH_e$ and the ΔpH in one experiment, and how the distribution of [$^3$H]5FU across the plasma membrane, expressed as the $5FU_{int}/5FU_{ext}$ ratio, relates to the ΔpH. The results in Figure 2A show that the more acid the extracellular pH, the larger the –ΔpH was across the cell membranes, i.e. the greater the difference from zero. Over the pH range of 5–8 there was a significant correlation ($P < 0.001$) and this was also unaffected by the incubation time (results not shown). The results in Figure 2B suggested that when the ΔpH became more negative (i.e. the –ΔpH increased), there was a trend towards a higher $5FU_{int}/5FU_{ext}$ ratio. The maximum $5FU_{int}/5FU_{ext}$ ratio in this experiment, approximately 2:1, was observed at a ΔpH gradient of –1. At more negative values of ΔpH, there was no further increase in the $5FU_{int}/5FU_{ext}$ ratio.

**Figure 2** Relationship between ΔpH across Lettre cell plasma membranes to (**A**) $pH_e$ and (**B**) $5FU_{int}/5FU_{ext}$. Results show a single representative experiment performed in duplicate after incubation of cells at 5 × $10^7$ cells/ml for 5 min with 0.1 mM 5FU, where pH gradient (ΔpH) = $pH_e - pH_i$

The mean data from several experiments in which the $5FU_{int}/5FU_{ext}$ ratio was compared with $pH_e$, $pH_i$ and the ΔpH at an extracellular 5FU concentration of 0.5 mM are shown in Figure 3. A point of inflection occurs in the graph of $5FU_{int}/5FU_{ext}$ vs $pH_e$ at or above a $pH_e$ of 6.0 (Figure 3A). From $pH_e$ 6–8 there is a highly significant negative linear correlation ($P$ = 0.002) with the $5FU_{int}/5FU_{ext}$ ratio (i.e. the ratio rises as $pH_e$ falls), but no further increase in $5FU_{int}/5FU_{ext}$ occurs below $pH_e$ 6.0. This results in a threefold decrease in the $5FU_{int}/5FU_{ext}$ ratio from $pH_e$ 6 to 8. When the $5FU_{int}/5FU_{ext}$ ratio was plotted against $pH_i$, a similar inflection was observed at *c.* $pH_i$ 6.8 (Figure 3B), and *c.* –0.8 for the graph of $5FU_{int}/5FU_{ext}$ vs ΔpH (Figure 3C). Again, the linear parts of the graphs in Figure 3B and C showed significant correlation coefficients ($P$ = 0.017 and $P < 0.001$ for $pH_i$ and ΔpH respectively). Similar correlations were seen at an extracellular 5FU concentration of 0.1 mM (results not shown). At 0.5 mM 5FU, the mean ± s.d. correlation coefficients in these experiments for the plot of $5FU_{int}/5FU_{ext}$ vs pH were 0.96 ± 0.03, 0.98 ± 0.04 and 0.88 ± 0.03 for $pH_e$, ΔpH and $pH_i$, respectively, and an ANOVA demonstrated that $pH_e$ and ΔpH were significantly better correlated than $pH_i$ with the $5FU_{int}/5FU_{ext}$ ratio ($P$ = 0.0003). It should be noted that $pH_i$ is not an independent variable as the measurement is dependent on the $pH_e$ value (see equation in Materials and methods).

© Cancer Research Campaign 1998

Figure 3 Dependence of the 5FU gradient ($5FU_{int}/5FU_{ext}$) on $pH_e$, $pH_i$ and $\Delta pH$ in Lettre cells in the presence of 0.5 mM 5FU. Results show the mean ± s.e.m. of 4–6 experiments, where pH gradient ($\Delta pH$) = $pH_e - pH_i$. The parameters are significantly correlated over the $pH_e$ range of 6–8; *P*-values are 0.002, 0.017 and 0.001 for $pH_e$, $pH_i$ and $\Delta pH$ respectively

Figure 4 Effect of glucose on the $5FU_{int}/5FU_{ext}$ ratio in Lettre cells and correlation with (A) $pH_e$, (B) $pH_i$ and (C) $\Delta pH$. Results show a representative experiment performed in duplicate using 0.5 mM 5FU in the presence (●) and absence (○) of 10 mM glucose, where pH gradient ($\Delta pH$) = $pH_e - pH_i$

### Alteration of constituents of the incubation medium

Two experiments were performed in which glucose (10 mM) was included in the incubation medium to cause intracellular acidification from glycolytically produced lactic acid. The 5FU concentration in the extracellular medium was 0.5 mM. Glucose decreased the uptake of DMO: the $DMO_{int}/DMO_{ext}$ ratio was about 2 with glucose and 3.5 without glucose, implying that intracellular acidification had taken place after glucose was added to the medium, since from the equation on page 6 $pH_i$ is proportional to $DMO_{int}/DMO_{ext}$. A representative example of the 5FU results is shown in Figure 4, in which the $5FU_{int}/5FU_{ext}$ ratio is plotted against $pH_e$ and $pH_i$. The $5FU_{int}/5FU_{ext}$ ratio did not rise to such a high plateau at acidic $pH_e$: the highest values in the two experiments were 1.6–1.7. Nevertheless, there was still an approximately linear fall in the $5FU_{int}/5FU_{ext}$ ratio over the pH range 6–8 (Figure 4A), whereas the $pH_i$ values were clearly shifted to lower values in the presence of glucose (Figure 4B) resulting in a decrease of the $-\Delta pH$ (Figure 4C).

The importance of the $Na^+$ gradient in this process was investigated by replacing $Na^+$ in the incubation medium with $K^+$. When the cells were incubated in the presence of 0.5 mM 5FU, there was no decrease in the maximum $5FU_{int}/5FU_{ext}$ ratio, nor in the pH gradient when two parallel experiments were compared in which $Na^+$ was present in the medium (results not shown).

### Effect of FCCP

FCCP depolarizes the plasma membrane making it permeable to $H^+$ and uncouples oxidative phosphorylation. The effect on 5FU uptake over the $pH_e$ range 5–8 is shown in Figure 5. The addition of 10 μM FCCP during the 5-min incubation reduced by 35% the $5FU_{int}/5FU_{ext}$ ratio at pH 5, 6 and 7. In addition, there was a small

© Cancer Research Campaign 1998

**Figure 5** Effect of FCCP on the $5FU_{int}/5FU_{ext}$ ratio in Lettre cells. Results show the mean ± sem of eight experiments using 0.5 mM 5FU over the $pH_e$ range of 5–8 where ● is control and ■ 10 μM FCCP. $^{**}P < 0.01$, $^{*}P < 0.05$ compared with FCCP-treated (paired *t*-test)

but significant decrease in the $pH_i$ of 0.12 at $pH_e$ 6 and 7, while both the intra- and extracellular [$Na^+$] and [$K^+$] were unchanged by FCCP treatment at all $pH_e$ values. In four of these eight experiments, the total intracellular [ATP] was determined to be 1.0 ± 0.1 μmol $g^{-1}$ (mean ± sem), and FCCP caused a mean decrease in this concentration of 33%.

### Distribution of 5FU across HT29 colon adenocarcinoma plasma cell membranes

Similar results to those found in the Lettre cells were also found when human HT29 cells were incubated with 5FU (Figure 6). Using the same $pH_e$ range of 5–8, the maximum recorded ΔpH was –1.5 (at $pH_e$ 5), resulting in a mean $5FU_{int}/5FU_{ext}$ ratio of 1.66. This ratio was significantly different ($P < 0.05$) from that measured when the ΔpH was close to zero ($pH_e$ 7 or 7.5). When the $5FU_{int}/5FU_{ext}$ ratio was plotted against $pH_e$, there was a highly significant negative correlation over the $pH_e$ range 6–8 ($P = 0.0003$), similar to that found for Lettre cells.

### DISCUSSION

We have studied the pH dependence of 5FU uptake in two tumour cell lines: the human HT29, which has been used in $^{19}F$-MRS pharmacokinetic studies in vivo (McSheehy et al, 1997), and murine Lettre cells in which many plasma membrane studies have been performed (Bashford and Pasternak, 1984). Variation of the pH of the suspending medium ($pH_e$) was used to alter ΔpH, i.e. the difference between $pH_i$ and $pH_e$, so that a –ΔpH of 1.5–2.0 could be attained at $pH_e$ 5. An increase in the –ΔpH correlated with an increase in the $5FU_{int}/5FU_{ext}$ ratio, i.e. uptake of 5FU by the cells. This 5FU uptake by Lettre cells was markedly inhibited in the presence of the ionophore FCCP.

How can the relationship between 5FU uptake and pH be explained? In the absence of significant metabolism, the most obvious explanation would be that the drug is distributed across the cell membrane as a weak acid. The enolic hydroxyl groups of 5FU ionize as acids, but with a $pK_a$ of 8.1. If 5FU distributed like a weak acid, little intracellular accumulation would be expected under physiological conditions, because $pK_{5FU} > pH_i$. However, in

**Figure 6** Relationship between ΔpH across HT29 cell plasma membranes and (**A**) $pH_e$ and $5FU_{int}/5FU_{ext}$. Results show the mean ± sem from four experiments incubating $10^6$ cells $ml^{-1}$ for 5 min with 0.1 mM 5FU, where pH gradient (ΔpH) = $pH_e - pH_i$

the presence of 0.5 mM 5FU at a $pH_e$ of 6.0 and $pH_i$ of 6.7 (where the $5FU_{int}/5FU_{ext}$ of ≥2:1 was measured in the Lettre cells – see Figure 3), the calculated ratio would be about 1.0:1. At $pH_i$ 7.31 and $pH_e$ 6.63, which are the pH values more typical of physiological conditions, the calculated ratio would still only be 1.15:1. Thus, our observations cannot be explained simply on the basis that 5FU distributes as a weak acid.

Wohlhueter et al, (1980) showed that transport of 5FU into Novikoff rat hepatoma cells was inhibited by uracil, suggesting that 5FU uptake involved facilitated transport via the uracil transporter. They also showed that the initial rate of transport was dependent on pH and related to $pK_{5FU}$ as only the uncharged species is transported. The p*K* of uracil is 9.2, so it will be almost entirely in the uncharged form at all reasonable physiological pH values. 5FU, despite having a lower p*K* of 8.1, would be 90–95% in the uncharged form at around $pH_e$ 6.5–7.0 (the most likely $pH_e$ values to be encountered in tumours), whereas at a $pH_e$ of 6.0, 99% would be uncharged. In both cases, near-maximum initial rates of transport will be allowed, whereas at $pH_e$ 8.0, 50% of the 5FU would be charged and the initial rate of transport would be markedly reduced. These considerations do not explain the 'steady-state' 5FU distribution observed in the present experiments. This is because the

© Cancer Research Campaign 1998 

uracil transporter will transport the uncharged form of its 'ligand' equally efficiently either into or out of the cell, the amount of 5FU transported in either direction depending on the concentration of uncharged 5FU available on the inside and outside of the cell membrane. In our experiments, 5FU retention only occurs at pH values (intracellular or extracellular) in the range of 6–7, when the concentration of the uncharged species would not be significantly different from one side of the plasma membrane to the other. In addition, Wolhueter's work investigated initial rates of 5FU transport, whereas our experiments study an equilibrium, or steady state, that is unchanged for up to 20 min after incubation (Figure 1).

Yamamoto and Kawasaki (1981) studied facilitative transport in Ehrlich ascites cells (the cell line from which Lettre cells were derived) suspended in 5 mM glucose, and showed that 5FU uptake was directly coupled to the ATP derived from glycolysis. This active transport occurred in the absence of a $Na^+$ gradient. The initial rate was slightly stimulated by $Na^+$ (1.3-fold), suggesting that $Na^+$ here was acting as a co-factor and not in creating an electrochemical potential, as occurs in the active transport of glucose and many amino acids. They concluded that 5FU uptake in these cells resembled the ATP-driven, $Na^+$-independent amino acid transport that occurs in *Escherichia coli*. We also found that the $5FU_{int}/5FU_{ext}$ ratio was unaffected by the absence of $Na^+$ in the incubation medium, suggesting that in the steady state it was $Na^+$ independent.

An alternative explanation is that the electrochemical concentration gradient of $H^+$ that we have imposed across the plasma membrane, provides a proton motive force (p.m.f.) which can be used to perform transport work. The p.m.f. (or $\Delta p$) is related to the $\Delta pH$:

$$\Delta p = \Delta\Psi - (Z\Delta pH)$$

where Z is a constant of 59 mV at 37°C and $\Delta\Psi$ is the plasma membrane potential (Harrison and Lunt, 1980). Co-transport of 5FU may occur by proton symport, as long as sufficient p.m.f. exists, i.e. there continues to be a $-\Delta pH$ or the $\Delta\Psi$ is not depolarized. This hypothesis predicts that there would be no intracellular accumulation of 5FU in the absence of a $-\Delta pH$ across the cell membrane, or when the plasma membrane is depolarized. Figure 3C (Lettre cells) and Figure 6B (HT29 cells) show that the $5FU_{int}/5FU_{ext}$ ratio is indeed found to be 1.0 when the $\Delta pH$ across the cell membrane is zero, and, similarly, the $5FU_{int}/5FU_{ext}$ ratio is <1.0 when the cell membrane $\Delta pH$ becomes positive (i.e. $pH_e$ is more alkaline than $pH_i$). Table 1 shows the calculated p.m.f. for Lettre cells over the $pH_e$ range studied. Note that p.m.f. decreases by approximately two-fold from $pH_e$ 5 to $pH_e$ 8, which is a similar change in magnitude to that measured for the $5FU_{int}/5FU_{ext}$ ratio.

The hypothesis was tested experimentally by incubating Lettre cells with FCCP. FCCP is an ionophore which depolarizes plasma membranes, making them permeable to $H^+$, and uncouples mitochondrial oxidative phosphorylation. Under the conditions of our study, FCCP significantly decreased the $5FU_{int}/5FU_{ext}$ ratio by 35% at $pH_e$ 5–7, and the total intracellular [ATP] by a similar amount. As the ion gradients were unchanged (as assessed by the distribution of $K^+$ and $Na^+$ across the plasma cell membrane), we have hypothesized that the [ATP] was still sufficient to maintain ion gradients, and that the depolarization of the plasma membrane was responsible for decreasing the $5FU_{int}/5FU_{ext}$ ratio. This is consistent with the idea that 5FU uptake is dependent on p.m.f. However, these experiments are in no way conclusive evidence of our hypothesis, and further investigations are being pursued to distinguish between the depolarizing and uncoupling effects of FCCP.

**Table 1** Changes in $\Delta\Psi$ and p.m.f. in Lettre cells as a function of $\Delta pH$

| $pH_e$ | $\Delta pH$ | $\Delta\Psi$ (mV) | p.m.f. | $5FU_{int}/5FU_{ext}$ ratio |
|---|---|---|---|---|
| 5 | −1.66 | −4 | 99 | 1.97 |
| 5.5 | −1.23 | −15.5 | 86 | 2 |
| 6 | −0.76 | −27 | 70 | 1.96 |
| 6.5 | −0.44 | −38.5 | 64 | 1.62 |
| 7 | 0.06 | −50 | 47 | 1.13 |
| 7.5 | 0.05 | −61.5 | 59 | 0.95 |
| 8 | 0.29 | −73 | 57 | 0.71 |

Values for $pH_e$, $\Delta pH$ and the FU ratio ($FU_{int}/FU_{ext}$) are taken from the values in Figure 3 (mean of five experiments), while $\Delta\Psi$ is calculated from the data in Bashford and Pasternak (1984) in which for these cells, the $\Delta\Psi$ depolarizes by 23 mV for each unit decrease in the $pH_e$, when $\Delta\Psi = -57$ at pH 7.3. p.m.f. ($\Delta p$) is calculated from the equation: $\Delta p = \Delta\Psi - Z\Delta pH$, in which Z is a combination of constants equal to 59 mV.

Our results and hypothesis are consistent with the MRS observations of Guerquin-Kern et al (1991), who showed that more 5FU was retained in a rat fibrosarcoma at lower $pH_i$ (< 6.9), induced by glucose infusion, than at $pH_i$ 7.3, similar to the findings made in this study. Furthermore, Gerweck et al (1991) found that in tumours administration of glucose to the host induced a decrease in $pH_i$ but caused an even greater decrease in $pH_e$ 60 min later, thus increasing the $-\Delta pH$ still further. Thus, although $pH_e$ was not measured in the study of Guerquin-Kern et al (1991), it is possible that the increased retention of 5FU was due to an increased $-\Delta pH$ across the tumour cell membranes. The results of our experiments in which glucose was added to the medium, reported in Figure 4, are broadly in agreement with our hypothesis. Glucose lowered the $pH_i$, and thus decreased the $-\Delta pH$ (Figure 4C) and consequently the $5FU_{int}/5FU_{ext}$ ratio.

Prerequisite for 5FU cytotoxicity is intracellular metabolism of the prodrug to 5-fluoronucleotides, and thus the first step necessary for anti-tumour activity is 5FU uptake by the target cells. Furthermore, the therapeutic ratio will be enhanced if uptake into normal cells is minimized. Our results suggest ways in which both of these favourable effects occur with 5FU. At a lower $pH_e$, in which the $-\Delta pH$ is large, such as occurs in solid tumours, there would be an enhancement of 5FU uptake, whereas normal cells (in which $\Delta pH$ is usually positive) will tend to exclude 5FU. In solid tumours this could lead to an accumulation of 5FU relative to other normal tissues, which is precisely what has been recorded in biopsies (Peters et al, 1993) and also by non-invasive $^{19}F$ MRS in animal models and in the clinic (Findlay et al, 1993; Presant et al, 1994). The hypothesis suggests potential means of manipulating the tumour pH environment for therapeutic gain. For example, one of the many drugs used in combination with 5FU in the clinic is interferon α, which has been shown in vitro to increase activity of the plasma membrane $Na^+/H^+$ antiporter in human tumour cells leading to an increase in $pH_i$ (Maheshwari et al, 1991). Indeed, we have recently shown in HT29 tumours xenografted in nude mice that interferon α increased the $-\Delta pH$ via an increase in $pH_i$, and increased 5FU retention in these tumours (McSheehy et al, 1997).

### REFERENCES

Aschele C, Sobrero A, Faderan MA and Bertino JR (1992) Novel mechanism(s) of resistance to 5-fluorouracil in human colon cancer HCT-8 sublines following exposure to two different clinically relevant dose schedules. *Cancer Res* **52**: 1855–1864

© Cancer Research Campaign 1998

Au LS, Walker JS and Rustum Y (1983) Pharmacokinetic studies of 5-fluorouracil and 5′-deoxy-5-fluorouridine in rats. *J Pharmacol Exp Ther* **227**: 174–180

Bashford CL (1994) Measurement of ion fluxes and pH gradients across cell membranes. In *Methods in Molecular Biology*, 27: *Biomembrane Protocols: II Architecture and Function*, Graham JM and Higgins JA (eds), pp. 307–323. Humana Press: Totowa, NJ

Bashford CL and Pasternak CA (1984). Plasma membrane potential of Lettre cells does not depend on cation gradients but on pumps. *J Membrane Biol* **79**: 275–284

Bashford CL, Alder G, Micklem, KJ and Pasternak CA (1983) A novel method for measuring intracellular pH and potassium concentration. *Bioscience Reports* **3**: 631–642

Bergmeyer HU (1974) Methods of enzymatic analysis. Weinheim, Deutschland: Verlag Chemie.

Cohen JL, Irwin LE, Marshall GJ and Bateman JR (1982) Clinical pharmacology of 5-fluorouracil. *Cancer Chem Rep* **58**: 723–728

Dawson RMC, Elliot DC, Elliot WH and Jones KM (1969). *Data for Biochemical Research*, 2nd edn, pp. 484. Clarendon: Oxford, UK

El-Tahtawy A and Wolf W (1991) In vivo measurements of intratumoral metabolism, modulation, and pharmacokinetics of 5-fluorouracil, using $^{19}F$ nuclear magnetic resonance spectroscopy. *Cancer Res* **51**: 5806–5812

Findlay M and Leach MO (1994). In vivo monitoring of fluoropyrimidine metabolites: Magnetic Resonance Spectroscopy in evaluation of 5-fluorouracil. *Anticancer Drugs* **5**: 260–280

Findlay MP, Leach MO, Cunningham D, Collins DJ, Payne GS, Glaholm J, Mansi JL and McCready VR (1993) The noninvasive monitoring of low infusional 5-fluorouracil and its modulation by interferon-alpha using in vivo $^{19}F$ magnetic resonance spectroscopy in patients with colorectal cancer: a pilot study. *Ann Oncol* **4**: 597–602

Findlay MP, Raynaud F, Cunningham D, Iveson A, Collins DJ and Leach MO (1996) Measurement of plasma 5-fluorouracil by high-performance liquid chromatography with comparison of results to tissue drug levels observed using in vivo 19F magnetic resonance spectroscopy in patients on a protracted infusion with or without interferon-α. *Annals Oncol* **7**: 47–53

Gerweck LE, Rhee JG, Koutcher JA, Song CW and Urano M (1991) Regulation of pH in murine tumour and muscle. *Radiation Res* **12**: 206–209

Griffiths JR (1991) Are cancer cells acidic? *Br J Cancer* **64**: 425–427

Guerquin-Kern J-L, Leteurtre F, Croisy A and Lhoste J-M (1991) pH dependence of 5-fluorouracil uptake observed by in vivo $^{31}P$ and $^{19}F$ nuclear magnetic resonance spectroscopy. *Cancer Res* **51**: 5770–5773

Harrison R and Lunt GC (1980) *Biological Membranes, Structure and Function*, 2nd edn, pp. 184. Blackie: London

Heidelberger C, Chaudhuari NK, Danenberg P, Mooren D, Griesbach L, Duschinsky R, Schnitzer RJ, Pleven E and Scheiner J (1957) Fluorinated pyrimidines, a new class of tumour-inhibitory compounds. *Nature* **179**: 663–666

Heidelberger C, Danenberg PV and Moran RG (1983) Fluorinated pyrimidines and their nucleosides. *Adv Enzymol Rel Areas Mol Biol* **54**: 58–119

Hill SR and Bibby MC (1994) 5-Fluorouracil causes alterations in the pharmacokinetic profile of tauromustine in NMRI mice. *Cancer Chemother Pharmacol* **34**: 57–62

McSheehy PMJ, Maxwell RJ and Griffiths JR (1991) Detection of differential sensitivity to 5-fluorouracil in Ehrlich ascites tumour cells by $^{19}F$ NMR spectroscopy. *NMR Biomed* **4**: 274–278

McSheehy PMJ, Seymour MT, Ojugo ASE, Rodrigues LM, Leach MO, Judson IR and Griffiths JR (1997) A pharmacokinetic and pharmacodynamic study in vivo of human HT29 tumours using $^{19}F$ and $^{31}P$ magnetic resonance spectroscopy. *Eur J Cancer* **33**: (in press).

Maheshwari RK, Sidhu GS, Bhartiya D and Friedman RM (1991) Primary amines enhance the antiviral activity of interferon against a membrane virus: role of intracellular pH. *J Gen Virol* **72**: 2143–2152

Naguib FNM, el Kouni MH and Cha S (1985) Enzymes of uracil catabolism in normal and neoplastic human tissues. *Cancer Res* **45**: 5405–5412

Peters GJ, Lankelma J, Kok RM, Noordhuis P, van Groeningen CJ, van der Wilt CL, Meyer S and Pinedo HM (1993) Prolonged retention of high concentrations of 5-fluorouracil in human and murine tumours as compares with plasma. *Cancer Chemother Pharmacol* **31**: 269–276

Presant CA, Wolf W, Albright MJ, Servis KL, Ring III R, Atkinson D, Ong RI, Wiseman C, King M, Blayney D, Kennedy P, El-Tahtawy A, Singh M and Shani J (1990) Human tumour fluorouracil trapping: clinical correlations of in vivo $^{19}F$ nuclear magnetic resonance spectroscopy pharmacokinetics. *J Clin Oncol* **8**: 1868–1873

Presant CA, Wolf W, Waluch V, Wiseman C, Kennedy P, Blaynay D and Brechner RR (1994) Association of intratumoral pharmacokinetics of 5-fluorouracil with clinical response. *Lancet* **343**: 1184–1187

Stubbs M, Bhujwalla ZM, Tozer GM, Rodrigues LM, Maxwell RJ, Morgan FA, Howe FA and Griffiths JR (1992) An assessment of $^{31}P$ MRS as a method of measuring pH in rat tumours. *NMR Biomed* **5**: 351–359

Vaupel P, Kallinowski F and Okunieff P (1989). Blood flow, oxygen and nutrient supply, the metabolic microenvironment of human tumors: a review. *Cancer Res* **49**: 6449–6465

Wohlhueter RM, McIvor RS and Plagemann PGW (1980) Facilitated transport of uracil and 5-fluorouracil, and permeation of orotic acid into cultured mammalian cells. *J Cell Physiol* **104**: 309–319

Wolf W, Presant CA, Servis KL, El-Tahtawy A, Albright MJ, Barker PB, Ring III R, Atkinson D, Ong R, King M, Singh M, Ray M, Wiseman C, Blaynay D and Shani J (1990) Tumour trapping of 5-fluorouracil: in vivo $^{19}F$ NMR spectroscopic pharmacokinetics in tumour-bearing humans and rabbits. *Proc Natl Acad Sci USA* **87**: 492–496

Yamamoto S-I and Kawasaki T (1981) Active transport of 5-fluorouracil and its energy coupling in Ehrlich ascites tumour cells. *J Biochem* **90**: 635–642

© Cancer Research Campaign 1998